# Supplementary material for: Magnetic Fe2O3–SiO2–MeO2–Pt (Me = Ti, Sn, Ce) as Catalysts for the Selective Hydrogenation of Cinnamaldehyde. Effect of the Nature of the Metal Oxide
Source: Materials (Basel). 2019 Jan 29;12(3):413. doi: 10.3390/ma12030413 (PMC6384899; doi:10.3390/ma12030413)
Supplement: Supplementary file 1 [file materials-12-00413-s001.pdf]

materials

MDPI

*Supplementary Information*

# Magnetic $Fe_2O_3$–$SiO_2$–$MeO_2$–Pt (Me = Ti, Sn, Ce) as Catalysts for the Selective Hydrogenation of Cinnamaldehyde. Effect of the Nature of the Metal Oxide

**Robinson Dinamarca [1], Rodrigo Espinoza-González [2], Cristian H. Campos [1] and Gina Pecchi [1,3,*]**

[1] Depto. Físico-Química, Facultad de Ciencias Químicas, Universidad de Concepción, Edmundo Larenas 129, Concepción 4070371, Chile; robidinamarca@udec.cl (R.D.); ccampos@udec.cl (C.H.C.)
[2] Deparment of Chemical Engineering, Biotechnology and Materials, FCFM, Universidad de Chile, Beauchef 851, Santiago 8370456, Chile; roespino@ing.uchile.cl
[3] Millenium Nuclei on Catalytic Processes towards Sustainable Chemistry (CSC), Santiago 8340518, Chile
* Correspondence: gpecchi@udec.cl; Tel.: +56-41-220-3352

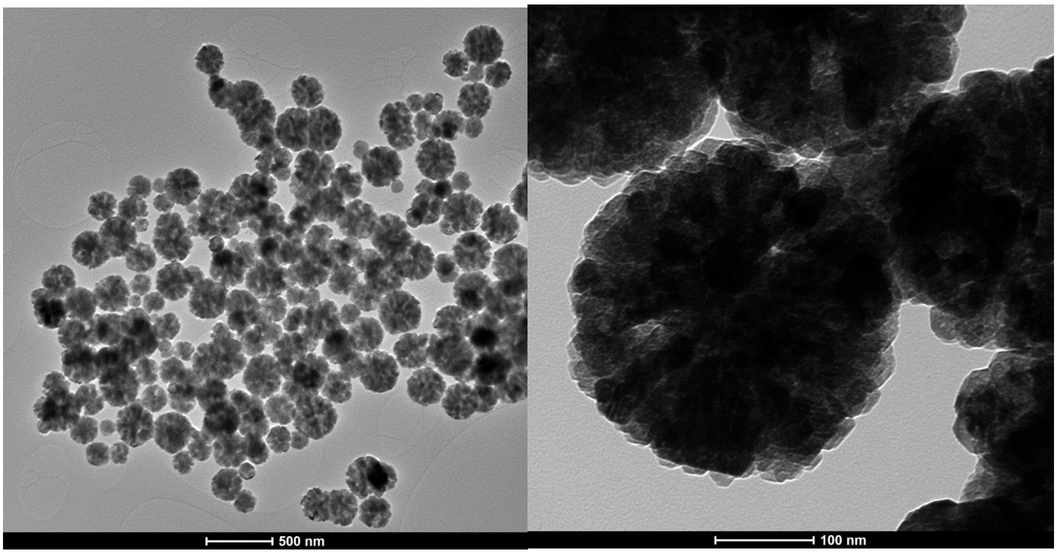


**Figure S1.** Micrograph of $Fe_3O_4$ nanoparticles.

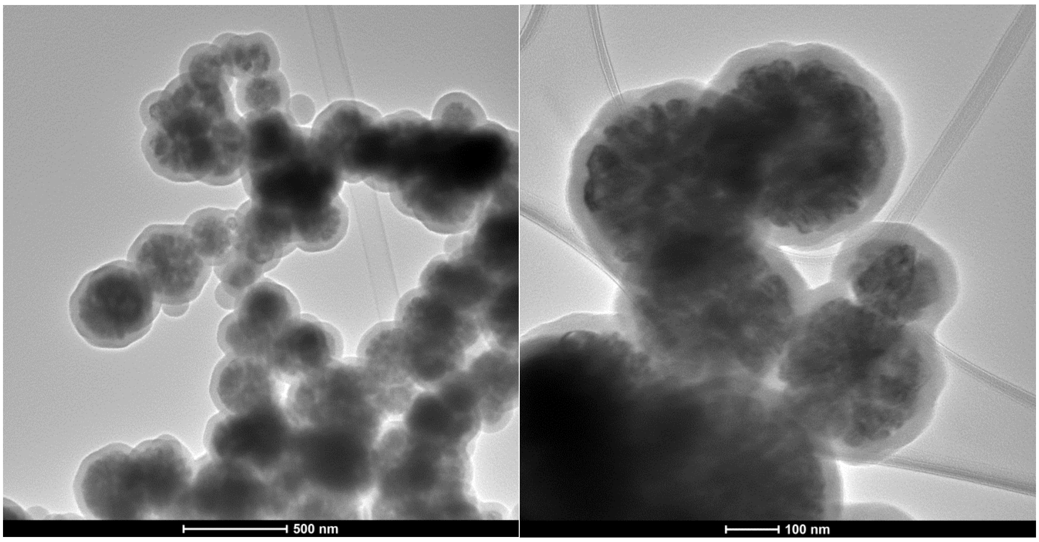


**Figure S2.** Micrograph of $Fe_3O_4$–$SiO_2$ *core-shell* material.

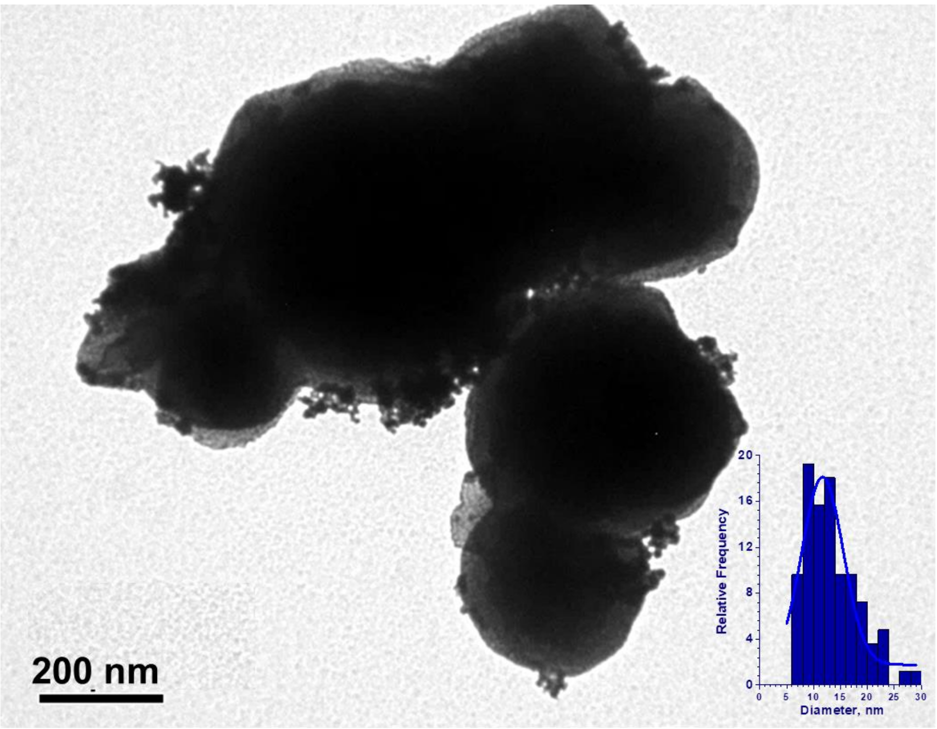


**Figure S3.** Micrograph of $Fe_2O_3$–$SiO_2$–$TiO_2$–5%Pt one-step *core shell* material.

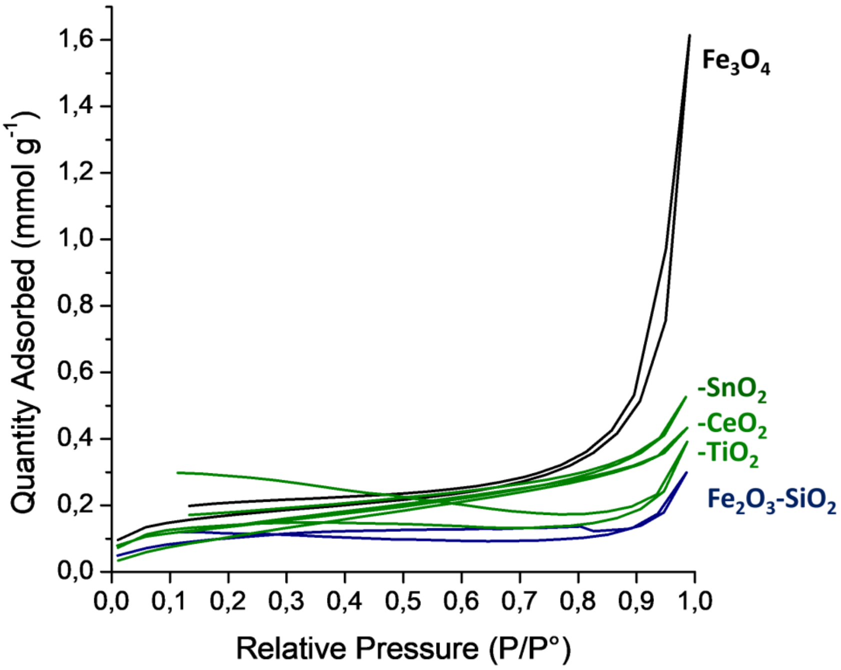


**Figure S4.** $N_2$ adsorption desorption Isotherm.

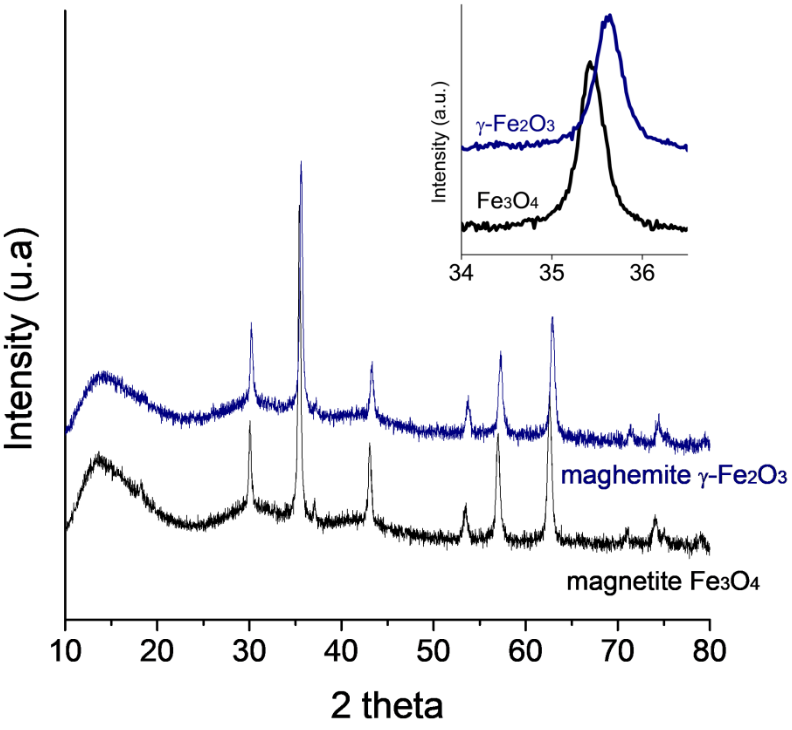


**Figure S5.** XRD patterns for $Fe_3O_4$ and $\gamma$-$Fe_2O_3$ nanoparticles.

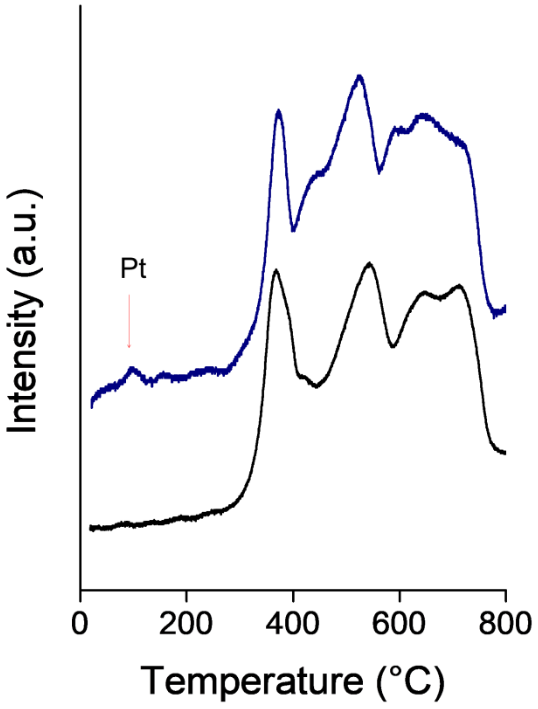


**Figure S6.** TPR-1 and TPR-2 of $Fe_3O_4$–$SiO_2$–$CeO_2$–Pt.
